# Fast Detection of SARS-CoV-2 RNA Directly from Respiratory Samples Using a Loop-Mediated Isothermal Amplification (LAMP) Test

**DOI:** 10.3390/v13050801

**Published:** 2021-04-29

**Authors:** Olympia E. Anastasiou, Caroline Holtkamp, Miriam Schäfer, Frieda Schön, Anna Maria Eis-Hübinger, Andi Krumbholz

**Affiliations:** 1 Institute for Virology, University Hospital Essen, University of Duisburg-Essen, 45147 Essen, Germany; Caroline.Holtkamp@uk-essen.de; 2 Labor Dr. Krause und Kollegen MVZ GmbH, 24106 Kiel, Germany; schaefer@labor-krause.de (M.S.); schoen@labor-krause.de (F.S.); krumbholz@labor-krause.de (A.K.); 3 Institute of Virology, Medical Faculty, University Hospital Bonn, 53127 Bonn, Germany; anna-maria.eis-huebinger@ukbonn.de; 4 Institute for Infection Medicine, Kiel University and University Medical Center Schleswig-Holstein, 24105 Kiel, Germany

**Keywords:** SARS-CoV-2, COVID-19, RT-PCR, nucleic acids, direct testing, loop-mediated isothermal amplification, LAMP

## Abstract

The availability of simple SARS-CoV-2 detection methods is crucial to contain the COVID-19 pandemic. This study examined whether a commercial LAMP assay can reliably detect SARS-CoV-2 genomes directly in respiratory samples without having to extract nucleic acids (NA) beforehand. Nasopharyngeal swabs (NPS, *n* = 220) were tested by real-time reverse transcription (RT)-PCR and with the LAMP assay. For RT-PCR, NA were investigated. For LAMP, NA from 26 NPS in viral transport medium (VTM) were tested. The other 194 NPS were analyzed directly without prior NA extraction (140 samples in VTM; 54 dry swab samples stirred in phosphate buffered saline). Ten NPS were tested directly by LAMP using a sous-vide cooking unit. The isothermal assay demonstrated excellent specificity (100%) but moderate sensitivity (68.8%), with a positive predictive value of 1 and a negative predictive value of 0.65 for direct testing of NPS in VTM. The use of dry swabs, even without NA extraction, improved the analytical sensitivity; up to 6% of samples showed signs of inhibition. LAMP could be performed successfully with a sous-vide cooking unit. This technique is very fast, requires little laboratory resources, and can replace rapid antigen tests or verify reactive rapid tests on-site.

## 1. Introduction

The ongoing pandemic caused by the *severe acute respiratory syndrome coronavirus* 2 (SARS-CoV-2) is a major challenge for humankind. The virus has infected more than 120 million people worldwide with a death toll of more than 2 million [1]. The impact of SARS-CoV-2 on healthcare systems and economies is huge. From the beginning of the pandemic, the demand for rapid, reliable, cheap, and easy-to-use diagnostics has been great and, even taking into account the progress made, there is still a big gap between supply and demand. This is especially true in low-resource settings where specialized equipment and personnel is often lacking [2].

Real-time reverse transcription polymerase chain reaction (RT-PCR) has been considered the gold standard for the detection of SARS-CoV-2 due to its high specificity and sensitivity [3]. It is, however, not without drawbacks, as it requires cost-intensive instruments (specialized equipment) and laboratory skills. Additionally, it is time-consuming, and the consumables are expensive, making it an unattractive option for low-resource settings. Furthermore, the reagents and materials required for RT-PCR are often scarce or unavailable, and as such, development and evaluation of alternative methods are urgently required [4]. In addition to the now widespread rapid antigen tests, loop-mediated isothermal amplification (LAMP) could be a useful alternative. This method can be performed without the use of thermocyclers and tends to be faster than RT-PCR. Several LAMP assays for SARS-CoV-2 detection have been developed [4], and a limited number of them have been evaluated using clinical samples with promising initial results [5,6,7,8,9,10].

There are various methods for detecting amplified nucleic acids (NA). Probe-based PCR assays are used most frequently. These require automated detection of the probe signal but enable specific detection and quantification of the amplificate in real-time during the run [11]. In contrast, conventional PCRs are rarely used for diagnostic purposes. After this type of PCR, the amplicon is usually detected by agarose gel electrophoresis based on its size and with the help of DNA intercalators. This procedure is less sensitive [12] and requires further evidence of specificity. The colorimetric LAMP enables direct visual detection of the amplicon by means of color change, which is quick, easy to perform, and independent of laboratory equipment. The generated amplicon may also be quantified by spectrophotometry as previously demonstrated [13].

Here we evaluate the performance of a commercially available LAMP assay for the detection of SARS-CoV-2 in respiratory tract samples compared to RT-PCR. Among other things, it is investigated to what extent the LAMP method is suitable for the direct detection of SARS-CoV-2-RNA without prior extraction of NA. Furthermore, the test results are compared depending on the sampling system used.

## 2. Materials and Methods

### 2.1. Loop-Mediated Isothermal Amplification for SARS-CoV-2 Detection

The performance of a research-use-only LAMP assay (SARS-CoV-2 Rapid Colorimetric LAMP Assay Kit, New England BioLabs, Ipswich, MA, USA) was tested under different conditions as indicated below. The LAMP reaction was conducted as recommended by the manufacturer. In brief, 2 µL of NA or 2 µL of the original respiratory material (a swab in viral transport medium, VTM, or a dry swab stirred in 500 µL of phosphate-buffered saline, PBS; 20 µL pre-incubated for 10 min at 99 °C) were pipetted together with 23 µL of LAMP reaction mix consisting of 12.5 µL of WarmStart Colorimetric LAMP 2X Master Mix (containing uracil-DNA glycosylase), 2.5 µL of SARS-CoV-2 LAMP primer mix (targeting parts of SARS-CoV-2 envelope (E) and nucleoprotein (N) genes), 5.5 µL of nuclease-free water, and 2.5 µL of guanidine hydrochloride in a 100 µL PCR reaction vessel. Guanidine hydrochloride has been shown to improve the performance of LAMP [14] and its use is thus recommended at a concentration of 40 mM. Since our VTM and the PBS did not contain guanidine hydrochloride, the protocol did not have to be modified. The primer design for the LAMP assay was described by Zhang et al. [14]. The primer sets used in this study were provided in the kit and sequence information of these primers is available in the instruction manual, which is available on the company’s website. The cDNA synthesis and the isothermal reaction were performed in one step. This is an advantage of the method, reducing sample handling time and decreasing the contamination risk. For each sample, a second separate vessel was prepared in order to demonstrate the presence of human NA, to assess if the reagents are active and used properly and thus demonstrate the absence of inhibition of reaction. For this purpose, 2.5 µL of the internal control LAMP primer mix (rActin) was used instead of the SARS-CoV-2 LAMP primer mix. The internal control LAMP primer mix targets actin RNA at an exon–exon junction, actin being the product of the human housekeeping gene, ACTB. Upon addition of the patient samples to the internal control or SARS-CoV-2 test reaction we verified that all starting reactions were pink, as a color change to yellow or orange at this stage would indicate that the patient samples were not compatible with the assay. Furthermore, a separate positive control (2 µL of SARS-CoV-2 positive control) and a non-template control (2 µL of nuclease-free water) were included in each experimental setting. The SARS-CoV-2 positive control (N gene) consists of a plasmid that contains the SARS-CoV-2 N gene (GenBank: MN908947.3). Thereafter, all vessels were incubated for 30 min at 65 °C in a thermocycler (unless otherwise stated) and immediately placed at room temperature for 5 min followed by recording the color of the reaction mixture. A yellow color was valued as positive while a pink color was valued as negative.

### 2.2. Deep Nasopharyngeal Swab Samples (NPS) Stirred in PBS

We tested 54 dry NPS for the detection of SARS-CoV-2 in the Labor Krause MVZ GmbH, Kiel, Germany. The swabs were stirred in 500 µL of sterile PBS without calcium and magnesium (Lonza, Basel, Switzerland) and tested for SARS-CoV-2 RNA using the LAMP assay and a triplex in-house RT-PCR designed for the detection of the SARS-CoV-2 N gene RNA as described before [15]. This RT-PCR uses primers and probes for the simultaneous detection of the SARS-CoV-2 N gene, Pseudomonas phi6 phage, and human glyceraldehyde-3-phosphate dehydrogenase. In the case of the RT-PCR, NA extraction was preferentially performed with the KingFisher Flex system and with the ABI7500 real-time thermocycler (both Thermo Fisher Scientific, Waltham, MA, USA) as described previously [15].

### 2.3. Deep NPS in VTM with and without NA Extraction for LAMP

We included 140 NPS for the detection of SARS-CoV-2 tested in the Institute for Virology at the Essen University Hospital. The swab collection kits contained VTM (Yocon virus sampling kit, Yocon Biology, Beijing, China), which was used for both the PCR and the LAMP as described above. Detection of the SARS-CoV-2 genomes via RT-PCR was performed using the RealStar^®^ SARS-CoV-2 RT-PCR kit 1.0 (Altona Diagnostics, Hamburg, Germany) on a CFX Connect Real-Time PCR detection system (Bio-Rad Laboratories, Hercules, CA, USA), and the NA extraction was performed using the MagNA Pure 96 System (Roche, Mannheim, Germany) according to the manufacturer’s instructions. MagNA Pure 96 System is an automated high-throughput NA isolation system which uses magnetic glass particle technology. For SARS-CoV-2 diagnostics, we used the viral NA SV Kit; 200 µL of VTM was pipetted into the MagNA Pure Processing Cartridges and further processed with the use of a binding/lysis buffer and proteinase K followed by an incubation step. The elution volume was 100 µL. In the case of 26 additional samples prior to performing LAMP, instead of heat inactivation, we applied the same NA extraction protocol as for the RT-PCR: 18 NPS were tested at the Institute for Virology, Essen, using the methods described above, while 8 NPS were tested at the Institute of Virology, University Hospital Bonn, after NA extraction (AltoStar AM16, Altona Diagnostics, Hamburg, Germany) with both LAMP and RT-PCR (Altona Diagnostics, Hamburg, Germany) on a CFX96 Deep Well Real-Time system.

### 2.4. Viral Load in Different RT-PCRs Used by the Laboratories

The number of RT-PCR cycles required for the fluorescent signal to exceed the background level (threshold) was recorded as threshold cycle (Ct). In order to achieve comparability between the results produced by the three laboratories, we tested two SARS-CoV-2 standard samples previously provided by INSTAND e.V. (Düsseldorf, Germany) as well as dilutions of them. The 10^4^, 10^5^, 10^6^, and 10^7^ SARS-CoV-2 copies/mL corresponded to Ct values of 31.4, 26.3, 23.1, and 20.2 (E-gene, Altona, Essen), 36.4, 32.8, 29.6, and 26.6 (N gene, in-house triplex RT-PCR, Kiel) and 29.5, 26.1, 22.9, and 19.3 (E gene, Altona, Bonn), respectively. Linear regression was performed for both series of values and the viral load in copies/mL was calculated based on an equation deriving from it.

To evaluate cross-reactivity, samples that tested positive by RT-PCR for endemic coronaviruses (HCoV NL63, HCoV OC43, HCoV 229E, and HCoV HKU1), influenza A virus, respiratory syncytial virus, human metapneumovirus, human rhinovirus, or human bocavirus 1, or several bacterial species (*Mycoplasma pneumoniae*, *Chlamydia pneumoniae*, *Haemophilus influenzae*, *Bordetella pertussis*, *Streptococcus pneumoniae*, *Streptococcus pyogenes, Staphylococcus aureus*, *Escherichia coli* and *Klebsiella pneumoniae*), were tested using the LAMP assay. Analyses for the above-mentioned viruses were performed by using the FTD Respiratory pathogen 21 assay (Fast Track Diagnostics, Mikrogen, Munich, Germany). Most samples containing viral pathogens were collected before the emergence of SARS-CoV-2. In the case of sampling from 2020 onwards, SARS-CoV-2 RT-PCR was negative. The viral samples were present in VTM, or in some cases, only as NA. For the bacterial samples, 100 µL of a SARS-CoV-2-free NPS stirred in 500 µL of PBS was spiked with a fresh colony of an ATCC or clinical strain of *Haemophilus influenzae, Bordetella pertussis, Streptococcus pneumoniae, Streptococcus pyogenes, Staphylococcus aureus, Escherichia coli* and *Klebsiella pneumoniae*, respectively. For *Mycoplasma pneumoniae* and *Chlamydia pneumoniae*, 10 µL inactivated and resolved external quality assessment samples were spiked into 90 µL of the NPS. All samples were mixed, heat-inactivated for 10 min at 99 °C (except for those only available as NA) and tested as described by LAMP. Amplification of rActin was noted as absence of inhibition.

Assessment of test samples for the improvement of diagnostic procedures has been approved by the ethics committees of the medical faculties of the University of Duisburg-Essen (20-9512-BO, 13 August 2020) and Kiel University (D467/20, 16 April 2020). Statistical analysis was performed using SPSS software (v23, SPSS Inc., Chicago, IL, USA), GraphPad Prism 6.0 (GraphPad, CA, USA), and the platform VassarStats (http://vassarstats.net) (accessed on 1 March 2021).

## 3. Results

### 3.1. Swabs in VTM: Moderate Sensitivity, Good Specificity, Frequent Inhibition

**Figure 1:**
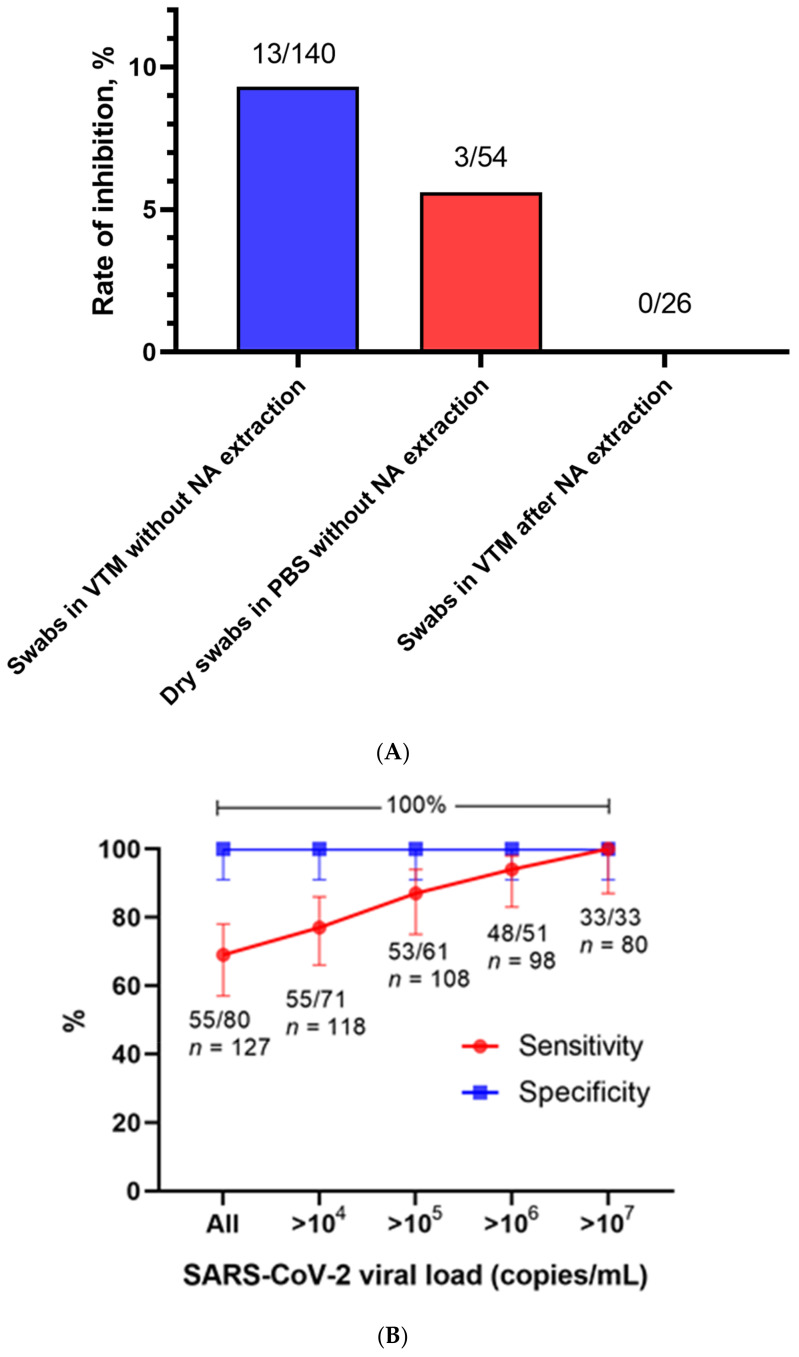

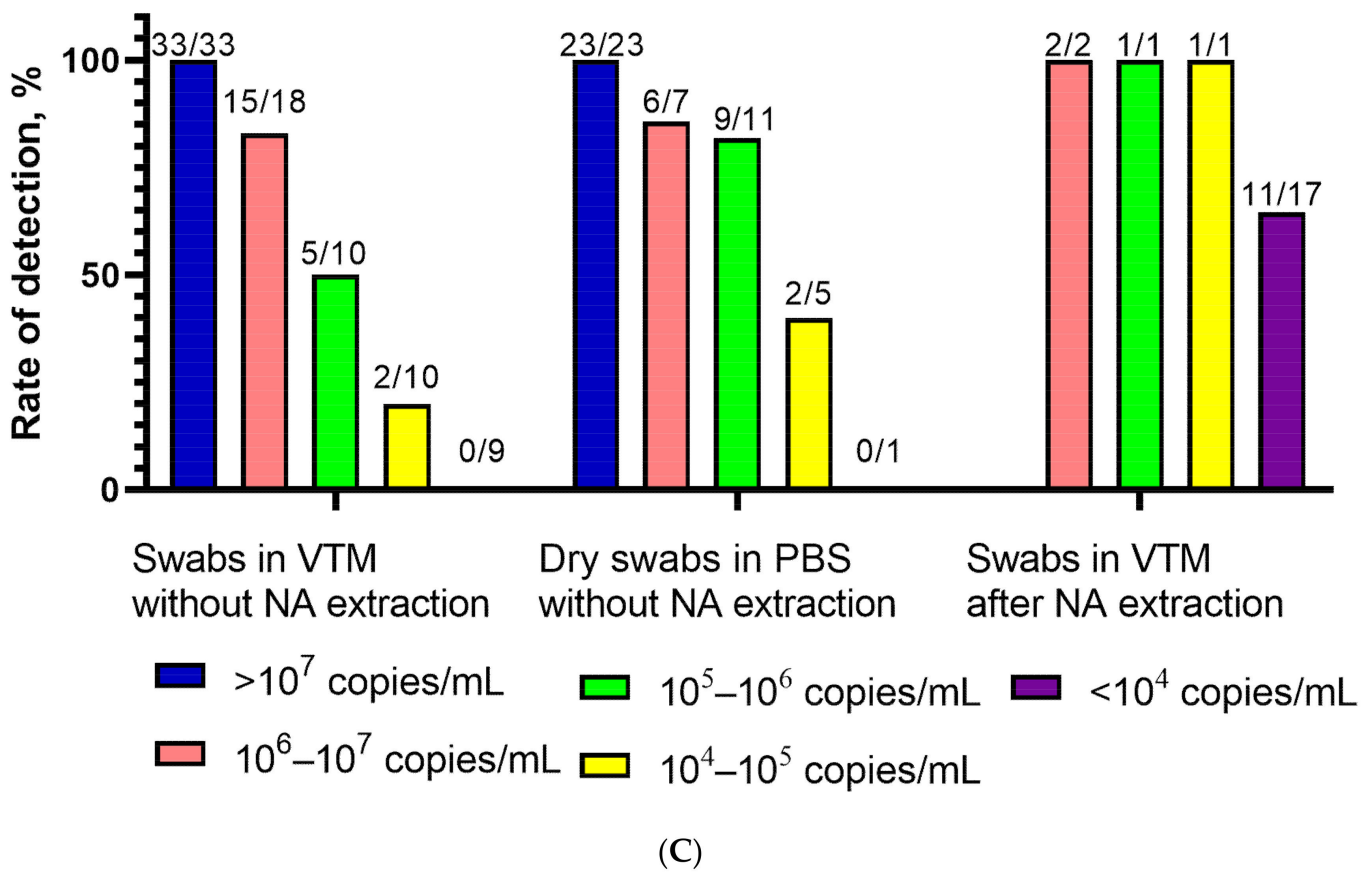
Inhibition rate and diagnostic performance of the loop-mediated isothermal amplification reaction (LAMP) assay. (**A**) The inhibition rate of the LAMP assay (i.e., failure in detection of rActin) was relatively high in nasopharyngeal swab samples tested without nucleic acid (NA) extraction. The numbers above the columns indicate the number of inhibited samples/number of samples tested using the LAMP assay. (**B**) The sensitivity and specificity of the LAMP assay without prior NA extraction was compared to RT-PCR after NA extraction (*n* = 140). Stratification was performed with the inclusion of all or a fraction of the samples according to their viral load in copies/mL. Inhibited samples were excluded. The sensitivity of the LAMP assay increased with increasing viral load. The specificity amounted to 100% irrespective of stratification. The error bars show the 95% confidence intervals. The numbers under the lines indicate the number of samples that tested positive using the LAMP assay/number of samples that tested positive using RT-PCR, *n* = number of negative and positive samples above the respective viral load threshold per RT-PCR. (**C**) The rate of detection differed amongst the three groups of samples. Dry swabs without NA extraction outperformed swabs in viral transport medium (VTM) without NA extraction. Performing NA extraction prior to testing increased the detection rate for samples with low viral loads considerably. The numbers above the columns indicate the number of samples that tested positive using the LAMP assay/number of samples that tested positive using RT-PCR.

We tested 140 consecutive NPS for the presence of SARS-CoV-2 RNA. The samples were acquired from the community through the public health department and included patients with possible/suspected SARS-CoV-2 infections based on clinical and/or epidemiological reasons. The samples were tested with RT-PCR after NA extraction (reference method) and LAMP after incubation at 99 °C for 10 min but without chemical extraction. Inhibition in LAMP was frequent (13 samples with no evidence of rActin signals, 9.3%) (Figure 1A). We observed no false positive results but had 25 (19.7%) false negative samples. True positive results could be observed in 55 (43.3%) samples and true negatives in 47 (37.0%), amounting to a sensitivity of 68.8% (CI 95% 57.3–78.4), a specificity of 100% (CI 95% 90.6–100), a positive predictive value of 1 (CI 95% 0.92–1), and a negative predictive value of 0.65 (CI 95% 0.53–0.76). A total of 64.7% (*n* = 55/85) of the samples were correctly identified as positive, while 5.9% (*n* = 5/85) of the positive samples showed signs of LAMP inhibition. The sensitivity of the method improved, when taking into account samples with an increasingly higher viral load (Figure 1B).

All samples (*n* = 33) with a viral load over 10^7^ copies/mL, 83.3% (*n* = 15/18) of the samples with a viral load between 10^6^ and 10^7^ copies/mL, 50.0% (*n* = 5/10) of the samples with a viral load between 10^5^ and 10^6^ copies/mL, and 20.0% (*n* = 2/10) of the samples with a viral load between 10^4^ and 10^5^ copies/mL were detectable with LAMP. None of the nine samples with a viral load less than 10^4^ copies/mL could be detected with LAMP (Figure 1C).

### 3.2. Dry Swabs in PBS: Higher Detection Rate in Samples with Low Viral Load Compared to Swabs in VTM

We tested 54 consecutive NPS for the presence of SARS-CoV-2 RNA. NPS were collected by dry swab and stirred in PBS as described above. The samples were tested with both RT-PCR after NA extraction, serving as the reference method, and LAMP after viral inactivation but without extraction. Three samples were inhibited (5.6%) (Figure 1A). All four SARS-CoV-2-free samples were correctly identified, while seven (14.9%, *n* = 7/47) of the positive samples were misidentified as false negative. Samples with a low viral load in the RT-PCR were more often undetectable with LAMP than samples with a high viral load. All samples (*n* = 23) with a viral load exceeding 10^7^ copies/mL were detectable, and nearly all samples (85.7%, *n* = 6/7 and 81.8%, *n* = 9/11) with a viral load between 10^6^ and 10^7^ and 10^5^ and 10^6^ copies/mL, respectively, were also detectable. The detection rate for samples with a viral load between 10^4^ and 10^5^ copies/mL fell to 40.0% (*n* = 2/5), while one sample with less than 10^4^ copies/mL was undetectable with LAMP (Figure 1C).

Additionally, we tested 18 samples (8 with a SARS-CoV-2 load of over 10^7^ copies/mL, and 10 negative for SARS-CoV-2) in parallel with a rapid lateral flow antigen test (NADAL^®^ COVID-19 Ag Test, Nal von Minden GmbH, Moers, Germany), with RT-PCR serving as the reference method. Both methods could correctly identify all samples as positive or negative. Furthermore, two false positive samples identified by another lateral flow antigen test (SARS-CoV-2 Rapid Antigen Test, Roche, Mannheim, Germany) were tested negative by LAMP following the 99 °C inactivation protocol.

### 3.3. Swabs in VTM after NA Extraction: Enhanced Detection Rate for Samples with a Low Viral Load, No Inhibition

We tested 26 NPS in VTM after NA extraction with both LAMP and RT-PCR. Fifteen (57.7%) samples tested positive in both methods and four (15.4%) samples tested negative in both methods. One sample (3.8%) tested positive in the LAMP test but not in the RT-PCR, while six (23.1%) tested positive in the PCR but not the LAMP. There was no evidence of LAMP inhibition for these samples (Figure 1A). All samples with a viral load higher than 10^4^ copies/mL and 64.7% (*n* = 11) of samples with a viral load less than 10^4^ copies/mL were detectable with LAMP (Figure 1C).

### 3.4. Sous-Vide and LAMP: A Thermocycler Is Not Required

**Figure 2:**
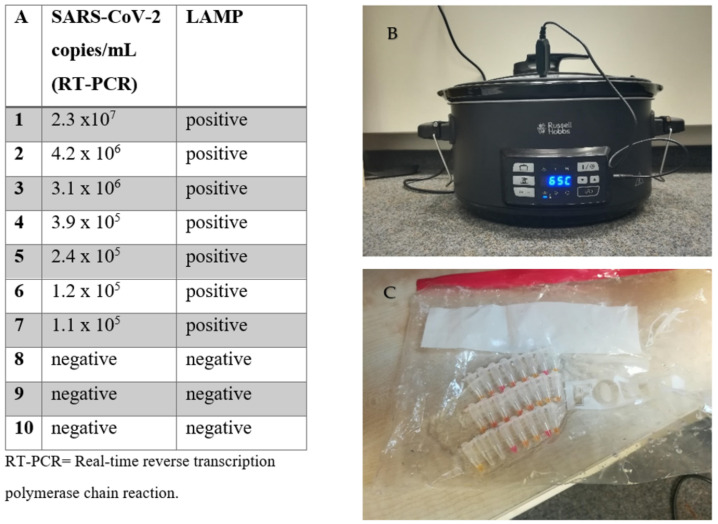
Loop-mediated isothermal amplification (LAMP) assay without a thermocycler. (**A**) Comparison of RT-PCR vs. the LAMP assay performed in a household sous-vide cooking unit. (**B**) Picture of the sous-vide cooking unit during testing, and (**C**) of the samples after completion of the assay.

We tested the performance of the LAMP assay using a commercially available sous-vide cooking unit for households (Russell Hobbs 25630-56 Sous-Vide, Russel Hobbs, Failsworth, UK). We used heat-inactivated samples (without NA extraction), including a total of three negative samples and seven SARS-CoV-2-positive samples. The samples were also tested using RT-PCR (RealStar^®^ SARS-CoV-2 RT-PCR kit 1.0) after NA extraction (MagNA Pure, Roche). The SARS-CoV-2 viral load of the positive samples ranged from 10^5^ to 10^7^ copies/mL. The samples were immersed in the unit, which was set at 65 °C for 30 min. During the 30-minute period, the sous-vide cooking unit managed to retain this temperature plus/minus 1 °C. Reaction vessels were placed into a plastic bag during amplification. All samples were correctly identified as positive or negative with the LAMP assay (Figure 2).

### 3.5. No Crossreactivity with Frequent Respiratory Viruses and Bacteria

Samples positive for endemic coronaviruses (HCoV NL63, HCoV OC43, HCoV 229E, HCoV HKU1), different influenza A virus types, respiratory syncytial virus, human rhinovirus, human metapneumovirus, human bocavirus-1 and nine bacterial species were tested with LAMP. None of these pathogens were detected by the LAMP assay. The results of the cross-reactivity evaluation of the viral pathogens are shown in Table 1 in more detail.

## 4. Discussion

Improving and expanding our diagnostic capacity in light of the COVID-19 pandemic is crucial. Several LAMP assays for SARS-CoV-2 detection have been developed [4], and a limited number of them have been evaluated using clinical samples with promising initial results [5,6,7,8,9,10]. A previous study presenting the performance of the LAMP assay indicated excellent specificity (99.5%) for NPS samples but moderate sensitivity (86%) when performing NA extraction [5]. Another study evaluating 106 leftover NPS showed that 59% could be correctly identified as SARS-CoV-2-positive by a LAMP assay without NA extraction, but the percentage went up to 82% when including samples with a viral load greater than 10^6^ copies/mL [16]. Performing LAMP using QuickExtract^®^ lysates directly prepared from crude patient respiratory tract swab samples led to the correct identification as positive of 50 to 64% of the samples, depending on the swab volume [8].

We tested 140 NPS samples in VTM, taken in a community setting through the public health department from individuals with possible/suspected SARS-CoV-2 infections due to clinical and/or epidemiological reasons. The LAMP assay carried out with VTM without NA preparation demonstrated excellent specificity (100%), but only moderate sensitivity (69%). Only 64% of the samples were correctly identified as positive, while 6% of the positive samples were inhibited. The detection rate of the method increased considerably for samples with a high(er) viral load. These results indicate that without NA extraction, the LAMP assay cannot compete with the combination of RT-PCR and NA extraction in terms of analytical sensitivity. However, it can reliably identify individuals with a high viral load, which is relevant when aiming to reduce transmission in a community setting.

We evaluated different test strategies in terms of both usability and diagnostic performance. Focusing on an NA extraction-free approach, we demonstrated that using dry swabs suspended in PBS led to a better detection rate for samples with lower viral loads compared to swabs in VTM. Compared to the PBS (without magnesium and calcium), VTM contains a number of additives. We suspect that these could interfere with direct testing of low viral loads. Previous studies have indicated that the addition of denaturizing agents, such as guanidine thiocyanate in the VTM, does not impact the detection of SARS-CoV-2 RNA via RT-PCR [17]. Using VTM with denaturizing agents helps maintain RNA integrity overtime and may decrease the risk of transmission of infectious agents in the laboratory [17]. The addition of guanidine thiocyanate increases the speed and sensitivity of LAMP [14] and is thus part of the NEB protocol (see Section 2). The use of denaturizing agents in VTM also has significant disadvantages. Media containing guanidine thiocyanate are not compatible with many testing platforms, as they can react with sodium hypochlorite, which is used for decontamination in many laboratories [18]. Furthermore, its use does not allow for cultures of viral isolates [17], which is a major disadvantage for both diagnostic (e.g., phenotypic drug resistance) and scientific purposes.

Previous studies have shown that dry swabs are appropriate for the PCR detection of respiratory viruses, including SARS-CoV-2 [19,20]. This is relevant, since using dry swabs could lower the cost of the sampling kit and provide an alternative in a setting where procuring VTM is difficult. Furthermore, viral isolation in cell culture is also successful with dry swabs stirred in PBS [15]. Unsurprisingly, implementing NA extraction prior to the performance of LAMP increased the analytical sensitivity of the method considerably compared to the extraction-free protocols.

The greatest advantage of LAMP compared to RT-PCR is that it can be carried out quickly. Direct testing of NPS takes around 45 min per sample. Furthermore, isothermal amplification can be readily implemented without use of specialized laboratory equipment as demonstrated in our study, making it an attractive option in low-resource settings, and in the case of RT-PCR reagents not being available. The LAMP assay is also scalable, allowing for the testing of a small number of samples up to high-throughput using 96-well thermoblocks. A disadvantage of the method is its limited analytical sensitivity when foregoing NA extraction. As shown above, performing NA extraction can significantly improve the performance of the method. In most laboratories, NA extraction is performed using (semi)-automated methods, which require specialized and expensive equipment. Manual extraction using column-based kits is time-consuming and requires centrifuges, making it unsuitable for POCT diagnostics. Thus, alternative NA extraction methods that are fast, reliable, and can be applied in the field may be an option for the improvement of the performance of LAMP. These include a 30 min silica coated magnetic bead NA method, which can be performed without the use of any electrical equipment [21], or the use of lysis and sample inactivation buffers such as QuickExtract (Lucigen) [8,22].

On-site testing with rapid SARS-CoV-2 antigen tests is assigned an important role in combating the COVID-19 pandemic. The greatest advantage of this kind of point-of-care (POCT) tests is their ease of use and the extremely short time span of 15 to 20 min until the test result is available. However, their performance varies markedly [9,23,24] and test sensitivities depend on the viral load, the time-point of sampling, and the presence of symptoms [23,25,26]. Importantly, although many POCTs demonstrate high specificity, false positive results have been reported [23,25,27]. Thus, positive POCT results should be confirmed by RT-PCT [28]. The analytical sensitivity and specificity of the LAMP assay is likely to be superior when compared with different POCTs [15,25,27], and largely meets the requirements of the World Health Organization [29], especially when using dry swabs. Because of these advantages, LAMP could replace rapid tests. If fast testing is in the foreground, it would alternatively be conceivable to use LAMP for on-site confirmation of reactive rapid tests. To do this, however, another NPS would have to be taken.

A strength of our study is the comprehensive evaluation of the LAMP assay for the detection of SARS-CoV-2 RNA under different conditions. Yet, our study also has limitations. Sampling was performed through different healthcare professionals, and as a result, a standardization beyond following the instructions given by the health authorities was not possible. Furthermore, the samples were tested in three laboratories using different RT-PCR assays. A comparison of the results with one another was made possible by using nationally available standard samples. Nevertheless, we believe that our results are relevant as they reflect the everyday testing routine and can be of use to the scientific community. The demand for SARS-CoV-2 diagnostics remains unabated and bottlenecks in resources are frequent. Expanding the diagnostic capacity and diversifying the diagnostic methods to fit different circumstances, for example, low-resource settings, seems prudent.

In conclusion, the LAMP assay demonstrated excellent specificity, but moderate sensitivity compared to RT-PCR. The isothermal amplification method can be implemented without the use of specialized laboratory equipment and may be useful for the replacement of POCTs or the immediate confirmation of reactive antigen tests.

## Figures and Tables

**Table 1:** Results of the loop-mediated isothermal amplification (LAMP) assay performed on respiratory samples positive for common pathogens.

Pathogen	Ct of the RT-PCR (If Available)	Number of Samples Tested Directly	Number of Samples Tested after Preparation of NA	Result of the LAMP for SARS-CoV-2
**HCoV HKU1**	18.6–28.5	4	-	negative
**HCoV NL63**	18.3–28	4	10	negative
**HCoV 229E**	17.1–27.8	3	-	negative
**HCoV OC43**	18.6–24.9	2	1	negative
**IAV H1N1**	18.8–24.3	3	-	negative
**IAV H3N2**	23.2	1	-	negative
**IAV not typed**	19.5–24.7	5	-	negative
**RSV**	18–22.3	12	-	negative
**RSV + HRV**	20.7–23 + 30.1–35	3	-	negative
**RSV + HBoV1**	18.8–20.6 + 35–35	2	-	negative
**HMPV**	15.3–23.6	3	3	negative
**HMPV + HRV**	24.5 + 20.2	1	-	negative
**HBoV1**	13.1–14.6	2	-	negative
***Haemophilus influenzae***	n.a.	1	-	negative
***Bordetella pertussis***	n.a.	1	-	negative
***Streptococcus pneumoniae***	n.a.	1	-	negative
***Streptococcus pyogenes***	n.a.	1	-	negative
***Staphylococcus aureus***	n.a.	1	-	negative
***Escherichia coli***	n.a.	1	-	negative
***Klebsiella pneumoniae***	n.a.	1	-	negative
***Mycoplasma pneumoniae***	n.a.	1	-	negative
***Chlamydia pneumoniae***	n.a.	1	-	negative

Ct: Cycle threshold; NA: nucleic acids; RT-PCR: reverse transcription real-time PCR; n.a.: not available; HCoV: human coronavirus; RSV: respiratory syncytial virus; HBoV1: human bocavirus-1; HMPV: human metapneumovirus; HRV: human rhinovirus; IAV: influenza A virus.

## Data Availability

The data presented in this study can become in part available on request from the corresponding author. The data are not publicly available due to data protection issues.
